# Luces y sombras de la inteligencia artificial en la medicina de laboratorio

**DOI:** 10.1515/almed-2025-0039

**Published:** 2025-03-03

**Authors:** Giuseppe Lippi, Mario Plebani

**Affiliations:** Servicio de Análisis Clínicos y Facultad de Medicina de la Universidad de Verona, Verona, Italy; Departamento de Medicina de Laboratorio, Universidad de Padua, Padua, Italia

**Keywords:** inteligencia artificial, medicina de laboratorio, deep learning, large language models

Aunque actualmente no existe una definición ampliamente aceptada de Inteligencia Artificial (IA), esta se suele describir como el empleo de sistemas informáticos para simular la inteligencia humana, con el propósito de realizar una extensa variedad de tareas que requieren razonamiento, aprendizaje y toma de decisiones [1]. El término “Inteligencia Artificial” fue acuñado por John McCarthy et al. a mediados de los años cincuenta, que la definieron como “la ciencia e ingeniería para desarrollar máquinas inteligentes que den muestras de pensamiento crítico comparable al de los humanos” [2]. Desde entonces, los notables avances tecnológicos han mejorado la potencia y las aplicaciones de las herramientas de IA, habiendo pasado esta a formar parte integral del ámbito personal y profesional de la población. Entre los principales componentes de la IA se incluyen el aprendizaje automático *(machine learning,* ML), que consiste en una serie de algoritmos diseñados para mejorar de forma autónoma mediante la experiencia, y el aprendizaje profundo *(deep learning,* DL), un subconjunto de herramientas de ML que emplean redes neuronales con múltiples capas para analizar datos complejos. Las propias redes neuronales son modelos computacionales que replican la estructura del cerebro humano y que cuentan con capacidades como el reconocimiento de patrones y el desarrollo de modelos predictivos [3]. La IA generativa, una rama de la IA, emplea modelos de DL y se centra principalmente en generar contenido nuevo, incluyendo textos (p.ej. *Large Language Models,* LLMs), imágenes y música, entre otros [3].

En el campo de la medicina, se han desarrollado sistemas de IA para mejorar la eficiencia y precisión, especialmente en tareas como el diagnóstico de patologías a partir de datos de imagen y el procesamiento de grandes volúmenes de datos clínicos y diagnósticos [4]. Debido al uso extendido de la tecnología y a su potencial para generar una gran cantidad de información clínica (llamada *“big data”)* en forma de resultados analíticos, la IA ha ido ganando impulso en el campo de la medicina de laboratorio [5]. Una encuesta reciente de la Federación Europea de Química Clínica y Medicina de Laboratorio (EFLM) reveló el elevado interés entre los profesionales de laboratorio en la formación en IA, aun cuando solo una minoría (aproximadamente el 25 %) de los laboratorios encuestados estaban inmersos en algún proyecto de IA [6].

Independientemente de las diferentes percepciones observadas entre los profesionales de laboratorio, resulta indiscutible que la IA seguirá desempeñando un papel cada vez más preeminente en la organización y actividad de los laboratorios médicos. En el momento actual, ya se están implementando varias herramientas de IA, como los modelos de ML para la optimización del procesamiento de muestras y gestión del control de calidad [7] y los módulos de verificación y validación automática integrados en el sistema informático del laboratorio (LIMS) [8], así como técnicas de morfología digital en hematología de laboratorio [9]. Además, los modelos de DL están siendo esenciales a la hora de manejar e interpretar los conjuntos de datos masivos generados en los estudios de investigación en el campo de la genómica y la proteómica.

Un ejemplo paradigmático de la forma en que la IA puede apoyar la toma de decisiones clínicas es el ensayo clínico aleatorizado simple ciego realizado recientemente en el que se asignó aleatoriamente a médicos de familia y a especialistas en medicina interna y urgencias para que emplearan los recursos tradicionales únicamente o tuvieran acceso a los mismos recursos complementados con LLM [10]. Aunque el empleo de LLM no mejoró de manera significativa el razonamiento clínico en comparación con el uso exclusivo de los recursos tradicionales (mediana de puntuación de razonamiento diagnóstico por caso: 76 % frente a 74 %; p=0,60), la mediana de puntuación de razonamiento diagnóstico del LLM empleado exclusivamente fue del 92 %, mejorando las puntuaciones de las dos cohortes de facultativos en más del 16 %.

Aunque cada vez está más asumido que la IA va a pasar a formar parte integral de la organización y las actividades de los servicios de laboratorio clínico de todo el mundo, aun quedan algunas dificultades por resolver (Tabla 1). El primer problema es la limitada flexibilidad de la IA a la hora de interpretar los datos analíticos. Frente a la cognición humana, que es capaz de emplear el juicio intuitivo y el razonamiento gestáltico [11], los sistemas de IA carecen todavía de la capacidad adaptativa necesaria para interpretar datos con el mismo nivel de comprensión contextual. El mismo principio sería aplicable al reconocimiento de imágenes. Aunque los modelos de DL se pueden entrenar para identificar imágenes digitales de tipos de células prototípicos, u otros componentes de los fluidos corporales, la heterogeneidad de presentación inherente a estos elementos, especialmente en lo relativo a la variabilidad de las alteraciones entre los distintos estados fisiológicos y patológicos, limita la precisión de la clasificación. Esta limitación persiste tanto si se emplean sistemas digitales de adquisición de imágenes celulares especializados, como si se emplean herramientas de IA más generales como ChatGPT [12]. En todos los casos, la generación actual de herramientas de IA seguirá precisando marcos de aprendizaje interactivos y la supervisión humana para la revisión de la clasificación inicial. De forma análoga, la interpretación de los síntomas humanos y los datos analíticos individuales por parte de la IA suele depender de una serie de algoritmos predefinidos diseñados para identificar patrones basados en datos y patologías existentes. No obstante, la presentación de las diversas patologías puede variar, pudiendo diferir también las posibles alteraciones analíticas asociadas a las diferentes patologías, lo que podría dificultar el suministro de interpretaciones en todo momento precisas o completas [5], 13]. Otras limitaciones son el exceso de dependencia de los sistemas de IA, que podrían sustituir completamente el razonamiento humano, su limitada flexibilidad y adaptabilidad, lo que podría llevar a errores a la hora de interpretar alteraciones analíticas complejas o poco frecuentes, tal como hemos ilustrado. Otras desventajas son la falta de armonización, dado que herramientas de IA distintas pueden dar respuestas diferentes a la misma cuestión clínica [14], las dificultades regulatorias que deben regir la integración de la IA en la práctica ordinaria de laboratorio, el coste de adquirir y mantener actualizado el *software*, la complejidad de la interacción entre los humanos y la IA, la falta de transparencia y justificación, debido al origen a menudo desconocido de los algoritmos, problemas de responsabilidades cuando se produce un error y los posibles sesgos en la toma de decisiones [15]. Finalmente, siempre será necesario emplear herramientas de validación externa para garantizar la reproducibilidad y generalización de toda herramienta o algoritmo de IA.

**Tabla 1:** Luces y sombras de la inteligencia artificial en la medicina de laboratorio.

Ventajas
– Posibilidad de agilizar procesos y actividades automatizando tareas habituales. – Capacidad para autovalidar y autoverificar datos analíticos, mejorando así la eficiencia y la precisión. – Asistencia en el reconocimiento de imágenes digitales para una mayor precisión diagnóstica. – Apoyo a la interpretación de datos clínicos, ayudando a la toma de decisiones clínicas. – Facilitar la redacción y edición de documentos. – Gestión y análisis de *big data*.

**Limitaciones**

– Flexibilidad y adaptabilidad limitadas a la hora de manejar datos inesperados y complejos. – Posibles errores a la hora de interpretar alteraciones analíticas complejas o infrecuentes. – Excesiva dependencia de la IA, lo que puede reducir el juicio clínico humano. – Falta de armonización, lo que puede derivar en la obtención de resultados distintos según la herramienta de IA utilizada. – Necesidad de regulación, lo que puede ralentizar la integración de la IA. – Costes elevados derivados de la implantación, mantenimiento y formación en el uso de sistemas de IA. – Complejidad en la interacción humano-IA. – Falta de transparencia y de justificación de los procesos de toma de decisiones mediante IA. – Falta de atribución de responsabilidades en caso de que se produzcan errores en la interpretación de los datos. – Consideraciones éticas y legales, incluyendo aspectos relacionados con la privacidad de los datos y sesgos en los algoritmos – Necesidad de validación externa para garantizar la reproducibilidad y generalización.

En conclusión, aunque se espera que la IA va a revolucionar la medicina de laboratorio mejorando su eficiencia, la precisión diagnóstica y la gestión de datos, esta también plantea una serie de desafíos sustanciales. La integración de la IA en la práctica habitual de laboratorio se debe plantear con cautela, resolviendo las dificultades más esenciales, como son el exceso de dependencia, la limitada flexibilidad y los posibles sesgos y errores. Por otro lado, es necesario también abordar otros aspectos de carácter regulatorio, ético y jurídico, con el fin de garantizar que la IA se despliega de forma transparenten donde se puedan atribuir responsabilidades. A medida que la IA va evolucionando, su importancia en los servicios de análisis clínicos irá creciendo. Sin embargo, para que su implementación sea satisfactoria, será necesaria la colaboración entre los profesionales de laboratorio, los desarrolladores tecnológicos, las autoridades regulatorias y los gestores sanitarios.
